# Evidence, Mechanism and Alternative Chemical Seedbank-Level Control of Glyphosate Resistance of a Rigid Ryegrass (*Lolium rigidum*) Biotype from Southern Spain

**DOI:** 10.3389/fpls.2017.00450

**Published:** 2017-03-29

**Authors:** Pablo T. Fernández-Moreno, Fernando Bastida, Rafael De Prado

**Affiliations:** ^1^Department of Agricultural Chemistry and Edaphology, University of CórdobaCórdoba, Spain; ^2^Department of Agroforestry Sciences, University of HuelvaHuelva, Spain

**Keywords:** rigid ryegrass, resistance, glyphosate, seedbank, target-site and non-target-site mechanisms

## Abstract

Rigid ryegrass (*Lolium rigidum*) is one of the most troublesome weeds in different crops in the Mediterranean region. A rigid ryegrass biotype from an olive grove in Jaén province (Andalusía, southern Spain), potentially resistant to glyphosate (RG), was tested for its resistance level through dose-response assays using a susceptible biotype (SG). To test the hypothesis of a non-target-site-based resistance, as point mutations are far less common mechanisms of glyphosate resistance, studies were also conducted to elucidate whether resistance was associated with biochemical, metabolism, molecular and/or physiological mechanisms. Alternative herbicide-based control options, including single-herbicide or herbicide mixtures with glyphosate, applied at seedling, tillering or full heading stages, were tested in field experiments for 2 years for their efficacy against rigid ryegrass plants and their effects on the soil seed bank. Resistance levels of the RG biotype were 23- (LD_50_) and 7-fold (GR_50_) higher compared to the SG biotype. The SG biotype exhibited a significantly greater shikimic acid accumulation than the RG one. At 96 HAT, 58 and 89% of applied ^14^C-glyphosate was up taken by leaves of RG and SG biotype plants, respectively, and, at this time, a significantly higher proportion of the glyphosate taken up by the treated leaf remained in its tissue in RG plants compared to the SG ones. The RG biotype did not reveal any point mutation in the glyphosate target site EPSP synthase. Overall, results confirmed reduced glyphosate uptake and translocation as being the mechanism involved in glyphosate resistance in the RG biotype. RG biotype responses to the alternative treatments tested *in situ* indicated that herbicide applications at the later growth stage tended to be less effective in terms of immediate effects on population size than earlier applications, and that only in some cases, the removal of at least 85% of the RG biotype was achieved. However, with few exceptions, the alternative treatments tested appeared to be highly effective in reducing the seed bank irrespective of the growth stage. The frequency of the resistant phenotype in the progeny of surviving plants of the RG biotype was dependent on treatment. Results suggest that a potential exists for effective management of glyphosate-resistant rigid ryegrass in olive groves in southern Spain.

## Introduction

Rigid ryegrass (*Lolium rigidum* Gaud.) is one of the most relevant weed problems in cereal and other grain crops, both in its Mediterranean area of origin (Izquierdo et al., 2003) and in south-western Australia (Holtum and Powles, 1991; Powles et al., 1998), where it was purposely introduced as a pasture plant (Kloot, 1983). Widespread and large populations of this weed are also characteristic of other crop types in the Mediterranean region, including fruit tree orchards, olive groves, and vineyards. Apart from being extensively cultivated in southern Australia, this plant species is also managed as a cover crop for reducing soil losses in erosion-prone crops, particularly olive groves in southern Spain (Alcántara-de la Cruz et al., 2016b). Rigid ryegrass is ranked among the weeds exhibiting most reported cases of herbicide resistance (Heap, 2017) and, currently, resistant populations appear to be highly frequent in surveyed countries, including Australia (Owen et al., 2007) and Spain (Loureiro et al., 2010), among others (Kaundun et al., 2011; Bostamam et al., 2012). First reports of herbicide resistance in this weed species date back to the early 1980s with ACCase- and ALS-inhibiting herbicides being involved (Heap and Knight, 1982). Thereafter, additional cases of resistance across different herbicide modes of action, and also multiple and cross resistance (Heap and Knight, 1986; Fernandez et al., 2016), have been repeatedly reported across 12 countries (Heap, 2017), including resistance to glyphosate. In fact, the first case of weed resistance to glyphosate was reported in 1996 for *L. rigidum*, in Australia (Powles et al., 1998).

Because of its broad control spectrum and fast degradation, glyphosate has become the most widely used herbicide since its introduction in 1974 (Duke and Powles, 2008; Powles and Yu, 2010). Glyphosate inhibits the enzyme 5-enolpyruvylshikimate-3-phosphate synthase (EPSPS) thus preventing biosynthesis of the aromatic amino acids, phenylalanine, tyrosine, and tryptophan, and of many secondary aromatic compounds (Amrhein et al., 1980). Inhibition of EPSPS leads to a rapid accumulation of shikimate and, eventually, to plant death (Shaner et al., 2012).

Resistance to glyphosate, currently identified in populations of 37 species worldwide (Heap, 2017), results from different mechanisms, generally classified as target-site and non-target-site resistance (NTSR) (Sammons and Gaines, 2014). Target-site resistance (TSR) can involve an EPSPS gene mutation or an over expression of the EPSPS enzyme. In the former case, the molecular basis for TSR was revealed to be a point mutation in the EPSPS gene consisting of a substitution at amino acid Pro-106 position by Ser, Thr, Ala, or Leu (Ng et al., 2005). As a result of this substitution, a decrease in the affinity of EPSPS for glyphosate binding is observed. This mutation has been reported in rigid ryegrass (Fernandez et al., 2015) and also in other weed species, including Italian ryegrass (Perez-Jones et al., 2007) and goosegrass (Baerson et al., 2002a). Recently, Yu et al. (2015) and Alcántara-de la Cruz et al. (2016a) reported glyphosate-resistant populations of goosegrass and hairy beggarticks, respectively, showing simultaneous Pro-106-Ser and Thr-102-Ile mutations. These are the first reports of a naturally evolving double mutation of the EPSPS gene in weeds, although it has been purposely used in transgenic maize (GA21) (Lebrun et al., 2003). While a single target-site mutation in the EPSPS gene seems to confer low levels of resistance to glyphosate in the order of two- to fourfold, the double mutation greatly increases resistance levels (Sammons and Gaines, 2014; Chen et al., 2015).

Amplified basal expression of EPSPS, also a TSR mechanism, has been found in glyphosate-resistant lines derived from the rigid ryegrass population in which glyphosate resistance was first described (Baerson et al., 2002b), and also in glyphosate-resistant horseweed (Dinelli et al., 2006) and hairy fleabane (Dinelli et al., 2008). Similarly, incremented basal EPSPS enzyme activity, associated with EPSPS gene amplification, has been reported as being a glyphosate-resistance mechanism in Italian ryegrass (Salas et al., 2012) and Palmer amaranth (Gaines et al., 2010). Amplified EPSPS expression provides additional active sites for PEP and S3P to bind normally and continue to move carbon flux through the shikimate pathway. For instance, resistant individual plants of Palmer amaranth had, on average, 77-fold more copies of the EPSPS gene, a 35-fold higher expression of EPSPS mRNA and an approximately 20-fold higher expression of EPSPS protein (Gaines et al., 2010).

Non-target-site resistance, which results from reduced glyphosate absorption and/or translocation, is far more common (Délye, 2013). As herbicide effects of glyphosate result from interference with the shikimate pathway, which is most active in meristematic tissues, translocation of the herbicide to these growing points must occur to a great extent. Glyphosate translocation takes place via phloem from treated leaves to sink meristematic tissues following sucrose movement. Phloem mobility of the glyphosate molecule is due to its unique combination of three acidity functions and one basic one. Any change in the structure of glyphosate that affects its zwitterionic characteristics reduces its ability to move through the plant (Shaner et al., 2012).

Unlike TSR, that only confers resistance to herbicides targeting the protein concerned, NTSR results in unpredictable resistance levels to different herbicides largely varying in their mode of action (Petit et al., 2010). Several studies have described NTSR in rigid ryegrass for up to 16 herbicide molecules with nine different action modes (Burnet et al., 1994). NTSR has also been described as being the most common mechanism of resistance to glyphosate (Powles and Yu, 2010), providing a 3- to 12-fold increase in resistance levels. Apart from rigid ryegrass (Burnet et al., 1994; Adu-Yeboah et al., 2014), NTSR mechanisms have also been reported in other *Lolium* species such as Italian ryegrass (Michitte et al., 2007; Nandula et al., 2008) and perennial ryegrass (Ghanizadeh et al., 2015), and in several other weed species, including johnsongrass (Vila-Aiub et al., 2012), sourgrass (de Carvalho et al., 2012), and horseweed (Koger and Reddy, 2005). On the other hand, rapid sequestration of glyphosate into vacuoles, leading to reduced amounts in the target-site, has been found in populations of *Conyza* and *Lolium* species. This mechanism has proved to confer a 14-fold increase in resistance to glyphosate (Ge et al., 2011, 2012). Enhanced metabolism to non-toxic, or less toxic, compounds including aminomethylphosphonic acid (AMPA), glyoxylate, sarcosine and formaldehyde has also been described as an underlying mechanism of glyphosate-NTSR (Gonzalez-Torralva et al., 2014). However, metabolism is not a frequent mechanism in weed resistance to glyphosate (Duke, 2011).

Jaén province (Andalusía, southern Spain) is the largest olive oil producer worldwide, contributing to approximately 20% of global annual production. Over recent years, farmers in this area have been experiencing increased difficulties in obtaining acceptable control levels of rigid ryegrass populations in olive groves under long-lasting glyphosate-based management schemes.

The ultimate success and sustainability of management practices is usually more determined by the long-term fate they impose on a weed population rather than by their effects on current population size (Mortensen et al., 2000).

On the basis of reported continuous lack of severe injuries following glyphosate applications at the labeled rate, we selected a population of rigid ryegrass from an olive orchard in Jaén province as a putative resistant biotype (RG). Experiments were conducted to (1) characterize response to glyphosate of the RG biotype relative to a susceptible biotype of rigid ryegrass, (2) determine the mechanisms involved in the lack of response, and (3) evaluate chemical control alternatives according to both immediate effectiveness in removing the standing RG biotype and longer-term effects through an ability to reduce its soil seed bank.

## Materials and Methods

### Plant Material

Mature seeds of a putative glyphosate-resistant (RG) rigid ryegrass biotype were collected in July 2013 from an olive grove, “El Álamo,” located in Beas de Segura, Jaén province, southern Spain. This olive grove had been treated with larger field doses than 1800 g ae ha^-1^ glyphosate for at least 11 consecutive years (Roundup^®^, 360 g ae L^-1^ as isopropylamine salt). Seeds of a susceptible (SG) ryegrass biotype were collected from a nearby olive grove that had never received glyphosate treatments. Seeds were stored for 3 months under laboratory conditions and thereafter germinated in Petri dishes with filter paper moistened with distilled water, placed in a growth chamber at 28/18°C (day/night), i.e., near optimal temperature conditions (Steadman et al., 2003), under a photoperiod of 16 h, 850 μmol m^-2^ s^-1^ photosynthetic photon flux, and 80% relative humidity. Resulting seedlings of RG and SG biotypes were transplanted into pots containing sand/peat in a 1:1 (*v/v*) ratio and placed in a growth chamber under the environmental conditions described.

### Glyphosate Whole Plant Dose-Response Assays

Herbicide treatments were applied at the 3–4 leaf growth stage. Glyphosate was applied in a laboratory chamber (SBS-060 DeVries Manufactering, Hollandale, MN, USA) equipped with 8002 flat fan nozzles delivering 200 L ha^-1^ at 250 KPa at the height of 50 cm. The following glyphosate (Roundup^®^ Energy SL, 450 g ae L^-1^ as isopropylamine salt, Monsanto) rates were used: 0, 31.25, 62.50, 125, 250, 500, 1000, 2000, and 4000 g ae ha^-1^. The experiment was designed using five replicates per rate. Plant mortality and dry mass were evaluated 21 days after the application (DAT). Dry mass was measured for aboveground parts of RG and SG plants after drying at 60°C for 72 h in a heater (J.P. Selecta S.A., Barcelona, Spain).

### Shikimate Accumulation in Leaves

The time patterns and extent of shikimate accumulation in glyphosate-exposed leaves of SG and RG rigid ryegrass plants were studied following two different spectrophotometric analyses. In the first analysis, 50 4-mm leaf disks were harvested from the youngest fully expanded leaf at the 3–4 tiller stage from 15 plants per biotype (Hanson et al., 2009). Five disks of fresh tissue were transferred to 2 mL eppendorfs containing 1 mL of 1 mM NH_4_H_2_PO_4_ (pH 4.4). One microliter of glyphosate was added to eppendorfs at the following concentrations: 0, 0.1, 0.5, 1, 5, 10, 50, 100, 200, 400, 500, 600, and 1000 μM. The eppendorfs were incubated in a growth chamber during 24 h under the above conditions. After 24 h, the eppendorfs were stored at -20°C until further analysis. Eppendorfs were removed from the freezer and thawed at 60°C for 30 min. Thereafter, 250 μL of 1.25 N HCL was added to each eppendorf. Again, they were introduced at 60°C for 15 min. A 125 μL aliquot from each eppendorf was pipetted into a new 2 mL eppendorf, and 500 μL of periodic acid and sodium metaperiodate (0.25% [wt/v] each) was added. After incubation at room temperature for 90 min, 500 μL of 0.6 N sodium hydroxide and 0.22 M sodium sulfite were added. Finally, the eppendorf’s content was transferred to glass vials. Samples were measured in a spectrophotometer at 380 nm within 30 min. For each glyphosate concentration and biotype, five replications were established and repeated twice.

In the second analysis, RG and SG plants at the 3- to 4-leaf stage were treated with glyphosate at 300 g ae ha^-1^ using the laboratory spray chamber and treatment conditions described above. At 24, 48, 72, and 96 h after treatment (HAT), 50 mg of plant tissue was harvested and placed in a vial containing 1 mL of 1 M HCl and then immediately frozen in liquid nitrogen. Shikimic acid accumulation was determined according to Singh and Shaner (1998). Sample absorbance was measured with a Beckman DU-640 spectrophotometer at 380 nm. Net shikimic acid accumulation was deduced from the difference between treated and non-treated plants in each biotype. The test was performed in triplicate on five treated and five non-treated plants per biotype.

### ^14^C-Glyphosate Uptake and Translocation

The assays were carried out according to Cruz-Hipolito et al. (2011). ^14^C-glyphosate (American Radiolabeled Chemicals, Inc., Saint Louis, MO, USA) was added to a commercial glyphosate to achieve a specific activity of 0.834 kBq μL^-1^. The final glyphosate concentration corresponded to 300 g ae ha^-1^ applied in 200 L ha^-1^. Plants of the RG and SG biotypes at the 3- to 4-leaf growth stage were treated with a drop of 1 μL (0.834 kBq plant^-1^) deposited with a micropipette (LabMate) onto the adaxial surface of the second leaf.

The treated leaf was washed with 3 mL of water: acetone (1:1 v/v) solution to remove non-absorbed ^14^C-glyphosate at 12, 24, 48, 72, and 96 h after drop application. The rinsate was mixed with 2 mL of scintillation cocktail and analyzed by liquid scintillation spectrometry (LSS) on a scintillation counter (Beckman LS 6500, Fullerton, CA, USA). The remainder of the plant was removed from the pot, and its roots were carefully washed with distilled water. The plant was divided into treated leaf, remaining shoot tissue, and roots. The plant parts thus obtained were dried at 60°C for 96 h and combusted in a Packard Tri Carb 307 biological sample oxidizer. Evolved ^14^CO_2_ was trapped and counted by LSS in a 18 mL mixture of Carbo-Sorb E and Permafluor E+ (1:1v/v) (PerkinElmer, Packard Bioscience BV). The amount of radiolabel deposited was checked by washing a treated leaf excised immediately after deposition. The experiment was arranged in a completely randomized design with five replicates and repeated twice. The mean radioactivity recoveries were 93.17 ± 2.48% and 95.86 ± 3.39% for RG and SG biotypes, respectively. The proportion of absorbed herbicide was expressed as [kBq in combusted tissue/(kBq in combusted tissue + kBq in leaf washes)] × 100.

### ^14^C-Glyphosate Visualization

Translocation of ^14^C-glyphosate in plants of RG and SG biotypes of rigid ryegrass was also visualized using a phosphor imager (Cyclone, PerkinElmer). Plants were treated and collected in the same way as described in the uptake and translocation assays. The whole plants were gently rinsed, pressed, and then left to dry at room temperature during 4 days. The dried plants were placed adjacent to a 25 cm × 12.5 cm phosphor storage film for 13 h and scanned for radiolabel distribution on a phosphor imager. Three plants were analyzed per biotype.

### Glyphosate Metabolism

Plants of RG and SG biotypes at the 3- to 4-leaf growth stage were treated with glyphosate at a rate of 300 g ae ha^-1^ as described in the dose-response assays section, while other plants were kept without treatment as non-treated controls. At 96 HAT, following the methodology described by Rojano-Delgado et al. (2010), glyphosate and its metabolites, i.e., aminomethylphosphonic acid (AMPA), glyoxylate, sarcosine, and formaldehyde, were determined by reversed polarity capillary electrophoresis using a G1600A Capillary Electrophoresis System (Agilent, Santa Clara, CA, USA) instrument equipped with a diode array detector (DAD, wavelength range 190–600 nm). Glyphosate, AMPA, sarcosine, formaldehyde, and glyoxylate were used as standards. Leaf tissues were washed with distilled water, flash-frozen in liquid nitrogen, and stored at -40°C until use. The aqueous background electrolyte consisted of 10 mM potassium phthalate, 0.5 mM hexadecyltrimethylammonium bromide, and 10% acetonitrile at pH 7.5. Calibration equations were established from non-treated plants and known concentrations of glyphosate and its metabolites, which were determined from their peak areas in the electropherogram. The average value for the content of glyoxylate naturally produced by the plant was subtracted from the average content of each biotype. The experiment was arranged in a completely randomized design with five replications per biotype and repeated twice.

### EPSPS Enzyme Activity Assays

The enzyme extraction was conducted according to the protocol described by Sammons et al. (2007). Five gram of the leaf tissue of RG and SG biotypes of rigid ryegrass plants were ground to fine powder in a chilled mortar. Immediately after that, the powdered tissues were transferred to tubes containing 100 mL of cold extraction buffer (100 mM MOPS, 5 mM EDTA, 10% glycerol, 50 mM KCl, and 0.5 mM benzamidine) containing 70 μL of β-mercaptoethanol and 1% in polyvinylpolypyrrolidone (PVPP). Samples were previously stirred and subsequently centrifuged for 40 min (18,000 × *g*) at 4°C. The supernatant was decanted into a beaker through a cheesecloth. (NH_4_)_2_SO_4_ was added to the solution to obtain 45% (w/v) concentration, with stirring during 30 min. After that, the mix was centrifuged at 20,000 × *g* for 30 min at 4°C. The previous step was repeated to precipitate the protein in the extracts but in that case with a (NH_4_)_2_SO_4_ concentration of 80% (w/v) stirring for 30 min. Finally, they were centrifuged at 20000 × *g* for 30 min at 4°C. All the pellets were dissolved in 3 mL of extraction buffer and dialyzed in 2 L of dialysis buffer (30 mm, 1000-MWC dialysis tubing at 4°C on a stir plate) over 12 h. The protein concentrations were determined by Bradford assay.

The assay for the determination of EPSPS activity followed the methodology described by Dayan et al. (2015) using the EnzCheck^®^ phosphate assay Kit (Invitrogen, Carlsbad, CA, USA) to determine the inorganic phosphate release. The EPSPS activity from biotypes was determined in the presence and absence of glyphosate. The glyphosate concentrations used were 0, 0.1, 1, 10, 100, and 1000 μM to determine the enzyme activity inhibition. The assay buffer used was composed of 1 mM MgCl_2_, 10% glycerol, and 100 mM MOPS, 2 mM sodium molybdate and 200 mM NaF. The experiment was repeated three times with three replications at each glyphosate concentration. EPSPS enzyme activity was expressed as percentage of enzyme activity in presence of glyphosate with respect to the control (without glyphosate). The EPSPS activity was calculated to determine the amount of phosphate (μmol) released μg of total soluble protein (TSP)^-1^ min^-1^.

### EPSP Synthase Sequencing

Total RNA was isolated from leaves using TRIzol reagent (Invitrogen, Carlsbad, CA, USA) according to the manufacturer’s instructions. RNA was then treated with TURBO DNase (RNase-Free; Ambion, Warrington, UK) to eliminate any DNA contamination and stored at -80°C. RNA integrity was verified in 0.8% agarose gel. The amount and quality of the RNA was measured by a NanoDrop ND-1000 spectrophotometer (Thermo Scientific, Walthman, MA, USA). First strand complementary DNA (cDNA) synthesis was started from the totality of the RNA adjusted to the same concentration in all the samples (50 ng μL^-1^). An iScriptTM cDNA Synthesis Kit (Bio-Rad Laboratories, Inc., Hercules, CA, USA) at a total reaction volume of 40 μL was employed following the manufacturer’s instructions. Primers (forward: 5′ AGCTGTAGTCGTTGGCTGTG 3′; reverse: 5′ GCCAAGAAATAGCTCGCACT 3′) were used and expanded a 543-bp fragment of the EPSPS gene that contains the mutation site described as conferring resistance to glyphosate in *Lolium* species. The PCR reactions were carried out using cDNA from 50 ng of total RNA, 1.5 mM MgCl_2_, 0.2 mM dNTP, 0.2 μM of each primer, 1 × buffer, and 0.625 units of a 100:1 enzyme mixture of non-proofreading (*Thermus thermophilus*) and proofreading (*Pyrococcus furiosus*) polymerases (BIOTOOLS, Madrid, Spain) in a final volume of 25 μL. All PCR reactions were in duplicate and cycling conditions were: 94°C for 3 min, 35 cycles of 94°C for 30 s, 55°C for 30 s and 72°C for 1 min, with a final extension cycle of 72°C for 10 min. An aliquot of the PCR product was loaded in a 1% agarose gel to check the correct band amplification. The rest of the PCR product was then purified using ExoSAP-IT^®^ for PCR Product Clean-Up (USB, Cleveland, OH, USA) as indicated by the manufacturer. Fifteen purified PCR products per biotype were sequenced (STAB VIDA, Caparica, Portugal). The resulting fragments were aligned and numbered based on published EPSPS sequences for rigid ryegrass [R rigid ryegrass from Fernandez et al. (2015); S rigid ryegrass from GenBank: AF349754].

### Chemical Alternatives to Glyphosate: Effects on the Standing Population and on the Soil Seed Bank

Field and laboratory/greenhouse experiments were conducted to evaluate the effectiveness of different alternative herbicide treatments in terms of immediate “*in situ*” control of the standing RG biotype of rigid ryegrass and of longer-term effects on soil seed bank size.

Three field trials were carried out during two consecutive seasons, 2013–2014 and 2014–2015. Single-herbicide or herbicide mixtures with glyphosate were applied either at early post-emergence (3- to 8-leaf stage, trial 1), at tillering (trial 2) or at full heading, immediately before flowering (trial 3) (**Table 1**). Three completely randomized blocks were established each study year within the cultivation row. Blocks were located within the cultivation row. The experimental unit was a plot of 2 m × 15 m. The herbicides were applied using a Pulverex backpack sprayer with a T coupling for the wand equipped with four flat fan nozzles, at a spraying pressure of 200 kPa, and calibrated to deliver a volume of 200 L ha^-1^. A strip of 1 m was established between plots within a block to prevent treatment overlap. Direct treatment effects on target plants were evaluated 60 days after application (DAT) in terms of percentage reduction of rigid ryegrass soil cover with respect to untreated plots, and treatment effectiveness was finally expressed as the complementary percentage.

**Table 1:** Herbicide treatments in rigid ryegrass in three different phenologic stages.

Early post-emergence, 3–8 leaves 07/03/2014 – 01/03/2015^a^			Tillering stage 08/04/2014 – 03/04/2015			Full heading stage 23/04/2014 – 29/04/2015		
Active ingredient^b^	HRAC group^c^	g ai ha^-1^	Active ingredient	HRAC group	g ai ha^-1^	Active ingredient	HRAC group	g ai ha^-1^
Control			control			control		
Glyphosate	G	1800^d^	Clethodim + glyphosate	A + G	100 + 1800	Glyphosate	G	1800
Clethodim	A	100	Cycloxydim + glyphosate	A + G	250 + 1800	Glufosinate	H	750
Cycloxydim	A	250	Quizalofop-*p*-ethyl + glyphosate	A + G	125 + 1800	Clethodim	A	100
Flazasulfuron	B	50				Cycloxydim	A	250
Flazasulfuron + glyphosate	B + G	50 + 1800				Quizalofop-*p*-ethyl	A	125
Quizalofop-*p*-ethyl	A	125				Diquat	D	800

*g ai ha^-1^= grams of active ingredient per hectare. ^a^ year 1 – 2; ^b^ glyphosate: Roundup Energy^®^, 45% p/v. SL, Monsanto, Spain; clethodim: Centurion Plus^®^, 12% p/v. EC, Bayer CropScience, Spain; cycloxydim: Focus Ultra^®^, 10% p/v. EC, BASF, Spain; flazasulfuron: Terafit^®^, 25% WG, Syngenta, Spain; quizalofop-*p*-ethyl: Master D^®^, 5% p/v. EC, Dow AgroSciences, Spain; glufosinate: Finale^®^, 15% p/v. SL, Bayer CropScience, Spain; diquat: Reglone^®^, 20% p/v. SL, Syngenta, Spain; ^c^ Abbreviations: HRAC, Herbicide-Resistance Action Committee; G, EPSPS inhibitors; A, ACCase inhibitors; B, ALS inhibitors; H, glutamine synthase inhibitors; D, PSI electron diverter;^d^ g ae ha^-1^= grams of acid equivalent per hectare.*

Apart from immediate effects on the abundance of the standing biotype, selected treatments were also evaluated according to their ability to reduce the soil seed bank on which future infestations would be dependent. To quantify treatment effects on seed bank size, we first estimated for surviving plants their mean seed set, i.e., the proportion of flowers produced by an individual plant transformed into single-seeded fruits (referred to as seeds throughout the paper). Rigid ryegrass plants of the RG biotype surviving the different herbicide treatments in the field trials were let to flower and fruiting. At maturity, inflorescences were bulk collected for each treatment within each block and stored in the laboratory for 3 months. As long as after-ripened rigid ryegrass seeds readily germinate under favorable laboratory conditions (Steadman et al., 2003), the seed set was measured as the germination fraction of floret units sampled from mature inflorescences. In each of the two study years, seed germination tests were carried out using 50 randomly chosen florets from each bulk collection. Floret samples were placed in plastic Petri dishes sealed with parafilm inside a growth chamber under the environmental conditions described above. Percentage of germination was recorded after 14 days. Seed set of surviving plants was established for the different treatments relative to seed set of untreated plants, and the resulting fractions were multiplied by the corresponding mean values of relative soil cover at 60 DAT to obtain relative measurements of the contribution of the standing population to the soil seed bank following exposure to the different herbicide treatments. The same as above, effectiveness in reducing the seed bank was finally expressed as the complementary percentages.

The frequency of the glyphosate-resistant phenotype among the progeny of plants of the RG biotype surviving the different “*in situ*” treatments was also estimated as follows. Seedlings resulting from germination tests were individually transferred to pots and placed in the greenhouse. At the 3- to 4-leaf stage, seedlings were treated with glyphosate at the labeled field rate (720 g ae ha^-1^) using the laboratory spray chamber described previously. Due to space limitations, the seedlings were divided into three lots per maternal treatment, and seedlings from each lot were simultaneously treated in the chamber. The number of plants surviving glyphosate treatment, i.e., exhibiting a resistant phenotype, was counted 21 days after treatments.

### Data Analysis

Whole plant dose-response and EPSPS enzyme activity data were subjected to non-linear regression analysis using a four-parameter log-logistic equation (Eq. 1) to determine the glyphosate dose causing 50% reduction in growth (GR_50_), 50% mortality (LD_50_), and inhibition of EPSPS activity by 50% (I_50_).

(1) $$Y=[(d\text{-}c)/(1+{\left(x/g\right)}^{b})]+c$$

where *Y* is above-ground weight or survival expressed as a percentage of the non-treated control, *c* and *d* are the parameters corresponding to the lower and upper asymptotes, *b* is the slope of the curve at the inflection point, *g* the herbicide rate at the inflection point (i.e., GR_50_, LD_50_, I_50_), and *x* (independent variable) is the herbicide rate.

Regression analyses were conducted using the *drc* package (Ritz et al., 2015) for the statistical environment R (R 3.2.4; R Core Team, 2015). Resistance indices were computed as RG-to-SG GR_50_, LD_50_, or I_50_ ratios. To test for a common GR_50_, LD_50_, or I_50_ for RG and SG biotypes, i.e., Resistance Index equal to 1, a lack-of-fit test was used to compare the model consisting of curves with biotype-specific *g* values with a reduced model with common *g* (Ritz et al., 2015).

Analysis of variance (ANOVA) was conducted using Statistix 9.0 (Analytical Software, USA) to test for differences between RG and SG biotypes in shikimate accumulation in leaves, either at 1000 μM glyphosate in the leaf segment or at 96 HAT in the whole plant analysis; proportion of the different glyphosate metabolites; proportion of applied ^14^C-glyphosate taken up by leaves, and proportions of absorbed ^14^C-glyphosate remaining in the treated leaf, translocated to roots and to the rest of the plant at 96 HAT; and basal enzyme activity. Percentage data were previously transformed (arcsine of the square root) to meet model assumptions of normality of error distribution and homogeneity of variance. Model assumptions were graphically inspected. When needed, differences between means were separated using the Tukey HSD test.

ANOVA was conducted in R accounting for the experimental design to test for the effects of herbicide treatment and year, and their interaction, on effectiveness in reducing soil cover of the R biotype in the field trials, and on seed set of plants surviving the treatments to fruiting. As above, response variables were previously transformed and model assumptions of homogeneity of variance and normality of errors were graphically inspected. Generalized linear mixed models (GLMM) with binomial error distribution and logit link function were used to test for treatment and year effects on the survival rate of the progeny from glyphosate in laboratory/greenhouse experiments. GLMMs allow for a proper treatment of hierarchical designs and, in the case of proportions, also they correct for varying initial sample sizes. GLMMs were conducted in R using the package lme4 (Bates et al., 2015). Model assumptions were graphically checked. For each maternal growth stage, we compared survival rates resulting from each maternal treatment to survival rates of untreated plants (the reference class), judging the effect as significant when the parameter of a specific treatment in the linear component of the model was significantly different from 0. Non-significant terms, starting with the interaction, were dropped one at a time and, in every step, the reduced model was compared with the previous model using likelihood ratio tests. We continued this process up to when no additional terms could be dropped.

## Results

### Glyphosate Whole Plant Dose-Response

**FIGURE 1:**
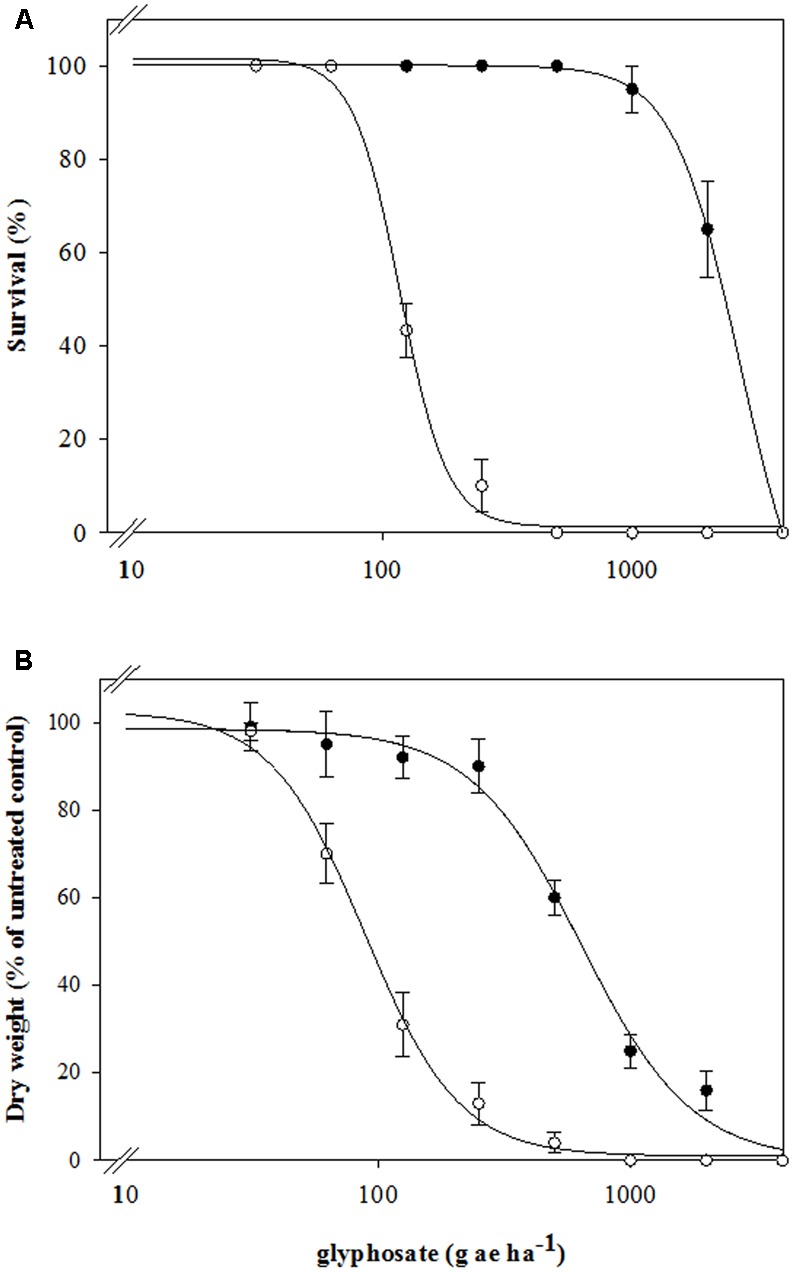
**Glyphosate dose-response on (A)** survival and **(B)** above-ground dry weight expressed as percentage of the mean untreated control of the R (•) and S (∘) biotypes of rigid ryegrass. Symbols denoted mean (*n* = 5) ± standard errors of the mean.

Dose-response assays clearly established differential susceptibility to glyphosate between the SG and the putative resistant RG biotypes of rigid ryegrass, both in terms of survival and aboveground dry biomass (**Figure 1** and **Table 2**). As expected, the SG biotype was highly susceptible to glyphosate and at the labeled field rate in Spain (720 g ae ha^-1^) it showed a very low survival and biomass (**Figure 1**). At this rate, however, 97% of the plants of the RG biotype survived the treatment, and aboveground dry biomass was only reduced to 48% of untreated plant biomass (**Figure 1**). The test for lack of fit, comparing a reduced model with common *g* parameter for SG and RG curves to a model with biotype-specific *g* values, was significant (*p* < 0.01) for both survival (LD_50_) and dry biomass (GR_50_), indicating that both rates do differ between biotypes and thus confirming resistance to glyphosate of the studied RG biotype from southern Spain (**Table 2**).

**Table 2:** Parameters of the log-logistic equations used to calculate the glyphosate rates required for 50% survival (LD_50_), reduction dry weight (GR_50_), or inhibit EPSPS activity (I_50_) of R and S biotypes of rigid ryegrass.

	Survival (%)					
	***c***	***d***	***b***	**LD_50_ (g ae ha^-1^)**	**RI**	***p***

R	-2.82	100.08	3.22	2712.05 ± 45.41	23.04	<0.0001
S	1.51	101.50	4.69	117.74 ± 4.54		

**Above-ground dry weight (%)**						

	***c***	***d***	***b***	**GR_50_ (g ae ha^-1^)**	**RI**	***p***

R	1.25	98.10	1.98	637.83 ± 32.90	7.17	<0.0001
S	1.41	102.41	2.38	88.87 ± 5.14		

**EPSPS activity (%)**						

	***c***	***d***	***b***	**I_50_ (μM)**	**RI**	***p***

R	1.14	100.59	1.65	8.23 ± 2.34	1.18	0.6034
S	1.29	101.36	1.96	6.94 ± 1.98		

*g ae ha^-1^ = grams of acid equivalent per hectare.*

### Shikimic Acid Accumulation in Leaves

**FIGURE 2:**
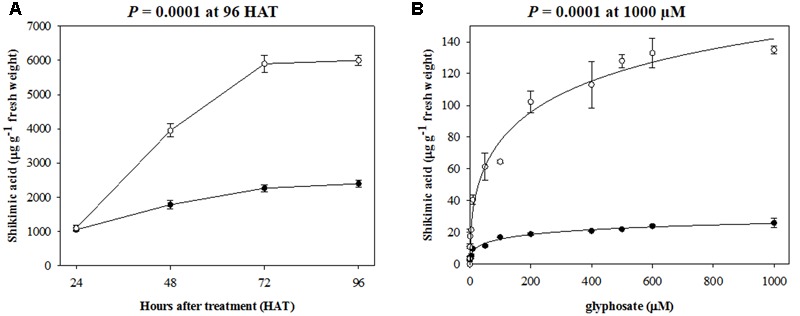
**Shikimic acid accumulation in (A)** plant leaves, and **(B)** leaf segments of plants from R (•) and S (∘) biotypes of rigid ryegrass. Symbols denoted mean (*n* = 5) ± standard errors of the mean.

Shikimic acid accumulation patterns in glyphosate-exposed leaves of rigid ryegrass plants of RG and SG biotypes are shown in **Figure 2**. In agreement with contrasting responses of RG and SG biotypes to glyphosate dose, leaves of SG plants accumulated much larger amounts of shikimate compared to RG plants, as demonstrated by the analyses at the whole plant and leaf segment levels. The whole plant analysis indicated that these differences in shikimate accumulation were already apparent 48 h after treatment and after 96 h, a highly significant (*p* < 0.001, DF = 1, *n* = 10) 2.5-fold greater shikimate concentration was found in leaves of the SG biotype (**Figure 2A**). The leaf segment analysis showed that increased shikimate levels in glyphosate-exposed SG biotype plant leaf segments were evident from very low glyphosate concentrations and at 1000 μM, the highest concentration tested, the difference was 5.2-fold greater in SG versus RG leaf segments (*p* < 0.001, DF = 1, *n* = 10) (**Figure 2B**).

### ^14^C-Glyphosate Uptake, Translocation, and Visualization

**FIGURE 3:**
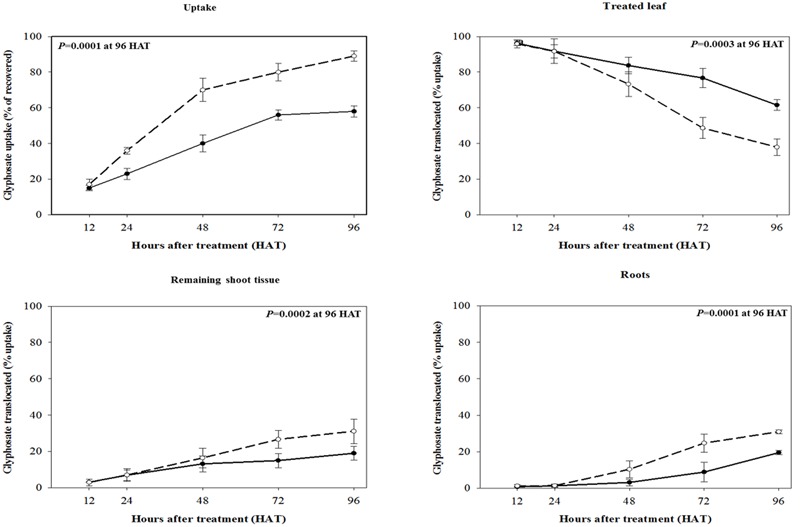
**^14^C-glyphosate foliar uptake and translocation to plant sections in R (•) and S (∘) biotypes of rigid ryegrass at 12, 24, 48, 72, and 96 h after glyphosate application.** Symbols denoted mean (*n* = 5) ± standard errors of the mean.

**FIGURE 4:**
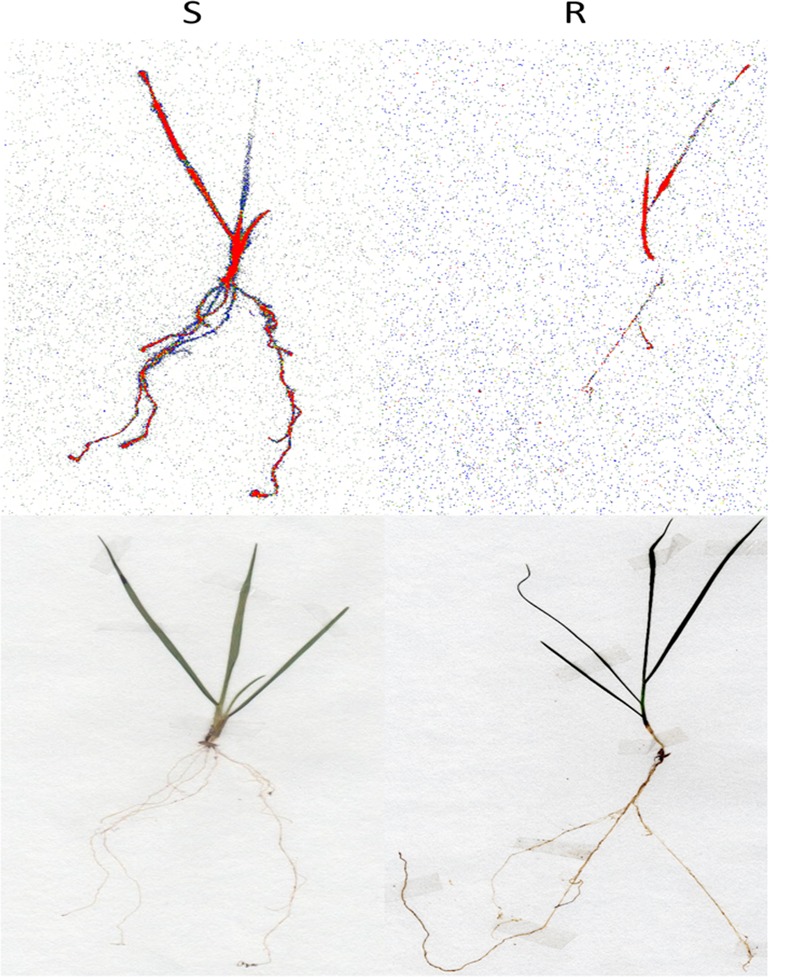
**Phosphorimaging visualization of ^14^C-glyphosate in plants of rigid ryegrass of susceptible (left)** and resistant **(right)** biotypes at 96 h after treatment.

The maximum ^14^C-glyphosate uptake for both biotypes was reached at 96 HAT, RG biotype rigid ryegrass plant leaves took up a significantly lower proportion (*p* < 0.001, DF = 1, *n* = 10) of applied ^14^C-glyphosate compared to the SG biotype, 58 and 89%, respectively (**Figure 3**). Differential patterns of glyphosate translocation within the plant were also evident between RG and SG biotypes. From 48 HAT onward, a higher proportion of the glyphosate taken up by the treated leaf remained in its tissues in RG plants compared to SG plants, and at 96 HAT, these proportions were significantly different (*p* < 0.001, DF = 1, *n* = 10), 61.6 and 37.9% for RG and SG plants, respectively (**Figure 3**). Accordingly, compared to the RG biotype, SG biotype plants showed a significantly higher proportion (*p* < 0.001, DF = 1, *n* = 10) of absorbed glyphosate in both the roots and rest of plant at 96 HAT (**Figure 3**). Overall, uptake and translocation assays indicated that glyphosate translocation was 2.4-fold greater in SG than in RG rigid ryegrass plants. Differences between rigid ryegrass biotypes in ^14^C-glyphosate translocation were also evidenced through phosphor imaging (**Figure 4**).

### Glyphosate Metabolism

A mean proportion of 87.4% of applied glyphosate remained un-metabolized at 96 HAT in rigid ryegrass plants with no significant differences (*p* = 0.8714, DF = 1, *n* = 10) in RG versus SG biotypes (**Table 3**). However, the remaining fraction was differentially metabolized to AMPA and glyoxylate in plants of RG and SG biotypes. Whereas AMPA was the main metabolism product of the RG biotype, it was glyoxylate for the SG biotype (**Table 3**).

**Table 3:** Glyphosate metabolism expressed as percentage of total glyphosate and their metabolites in R and S glyphosate biotypes of rigid ryegrass at 96 h after treatment.

Biotype	Glyphosate	AMPA	Glyoxylate
R	88.39 ± 3.15 A	8.59 ± 1.24 A	3.02 ± 0.14 B
S	86.41 ± 2.73 A	3.21 ± 0.91 B	10.38 ± 2.81 A
*p*	0.8714	0.0031	0.0024

*Means on a column followed by the same letter were not significantly different at α = 0.05. Mean values ± standard error of the mean. *AMPA*, aminomethylphosphonic acid. Treated with glyphosate at 300 g ae ha^-*1*^ at the 3–4 leaf growth stage.*

### EPSPS Enzyme Activity

The specific activity of EPSPS in the absence of glyphosate was similar in RG and SG biotypes (*p* = 0.824, DF = 1, *n* = 6), 0.0839 ± 0.0053 and 0.0781 ± 0.0093 μmol μg^-1^ TSP min^-1^, respectively. The concentration of glyphosate required to inhibit EPSPS activity by 50% (I_50_) was 8.23 and 6.94 μM in RG and SG biotypes, respectively, with no significant difference (*p* = 0.603) (**Table 2**).

### EPSPS Gene Sequencing

**FIGURE 5:**
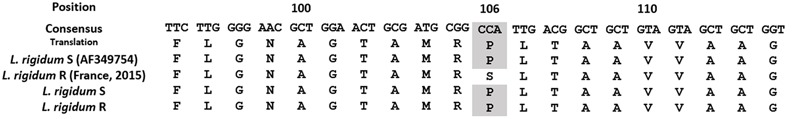
**Partial gene sequence alignment of the EPSP synthase of resistant (R) and susceptible (S) rigid ryegrass biotypes**.

The RG biotype of rigid ryegrass did not reveal any mutation at position Pro-106 in the EPSP synthase gene (**Figure 5**).

### Chemical Alternatives to Glyphosate: Effects on the Standing Population and on the Soil Seed Bank

**FIGURE 6:**
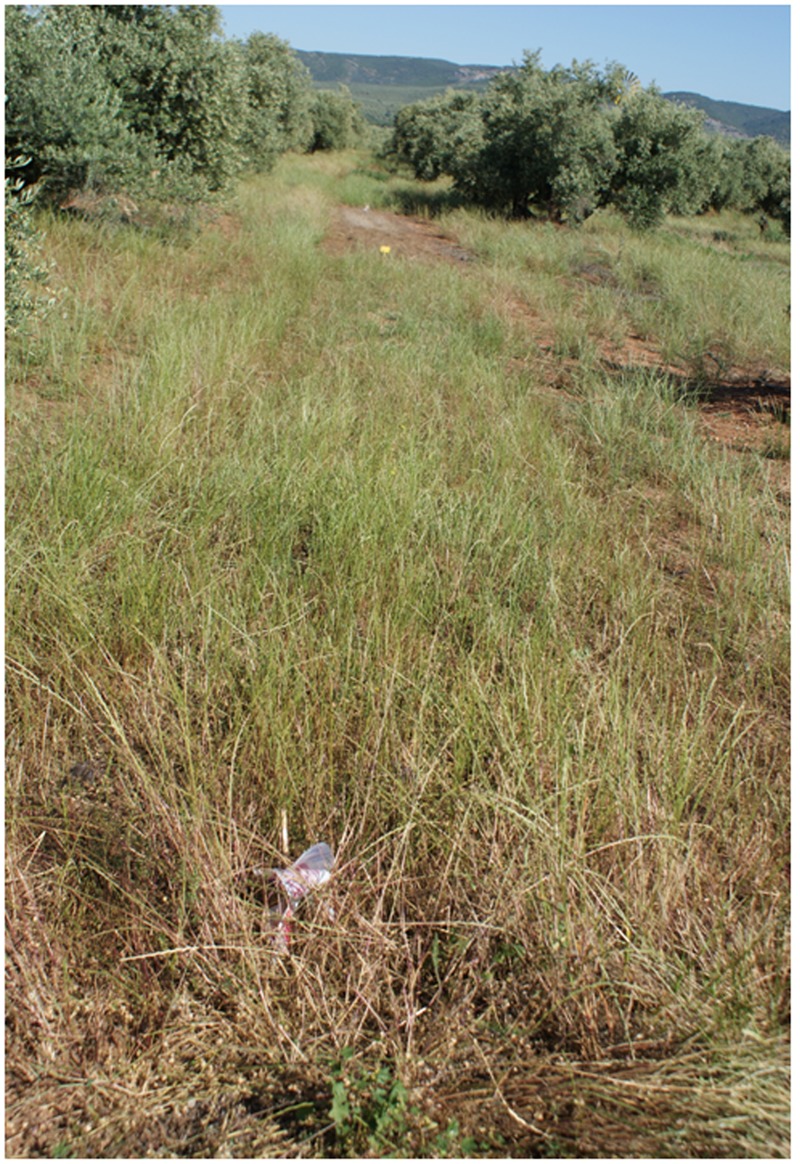
**Untreated rigid ryegrass**.

**FIGURE 7:**
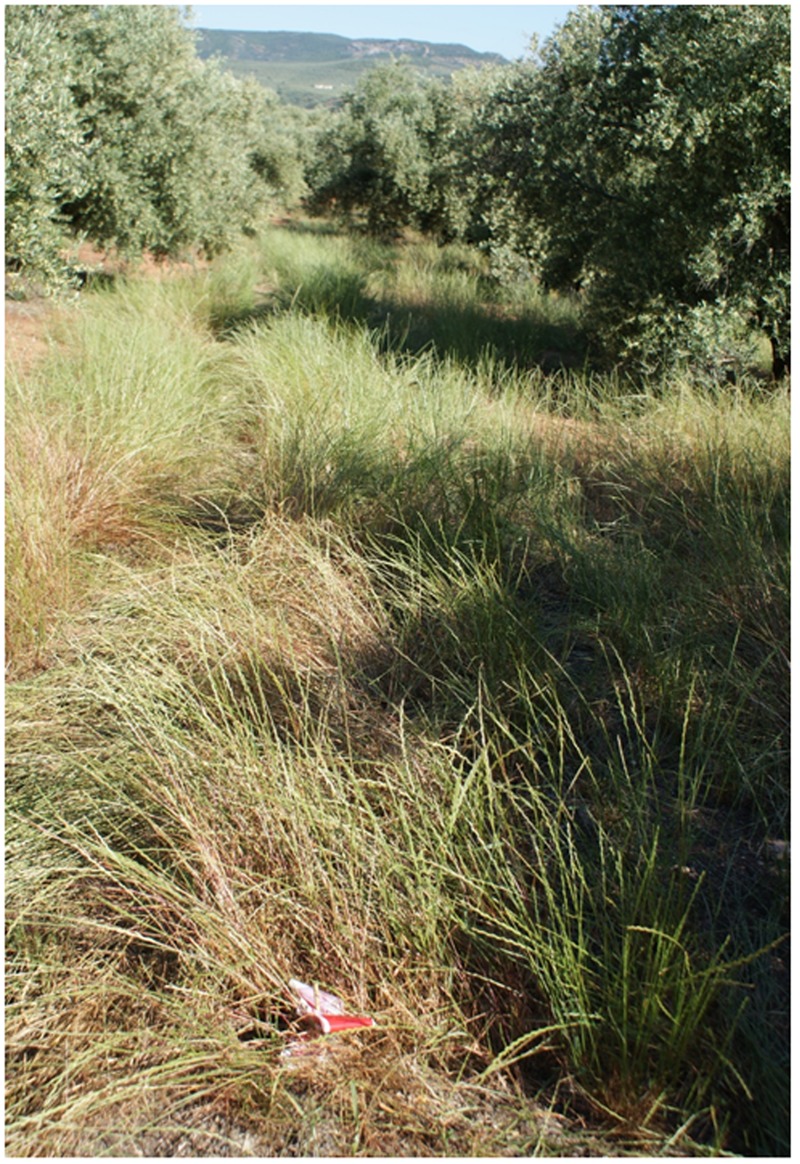
**Rigid ryegrass treated with 1800 g ae ha^-1^ (grams of acid equivalent per hectare) of glyphosate at early post-emergence and measured 60 days after treatment**.

**FIGURE 8:**
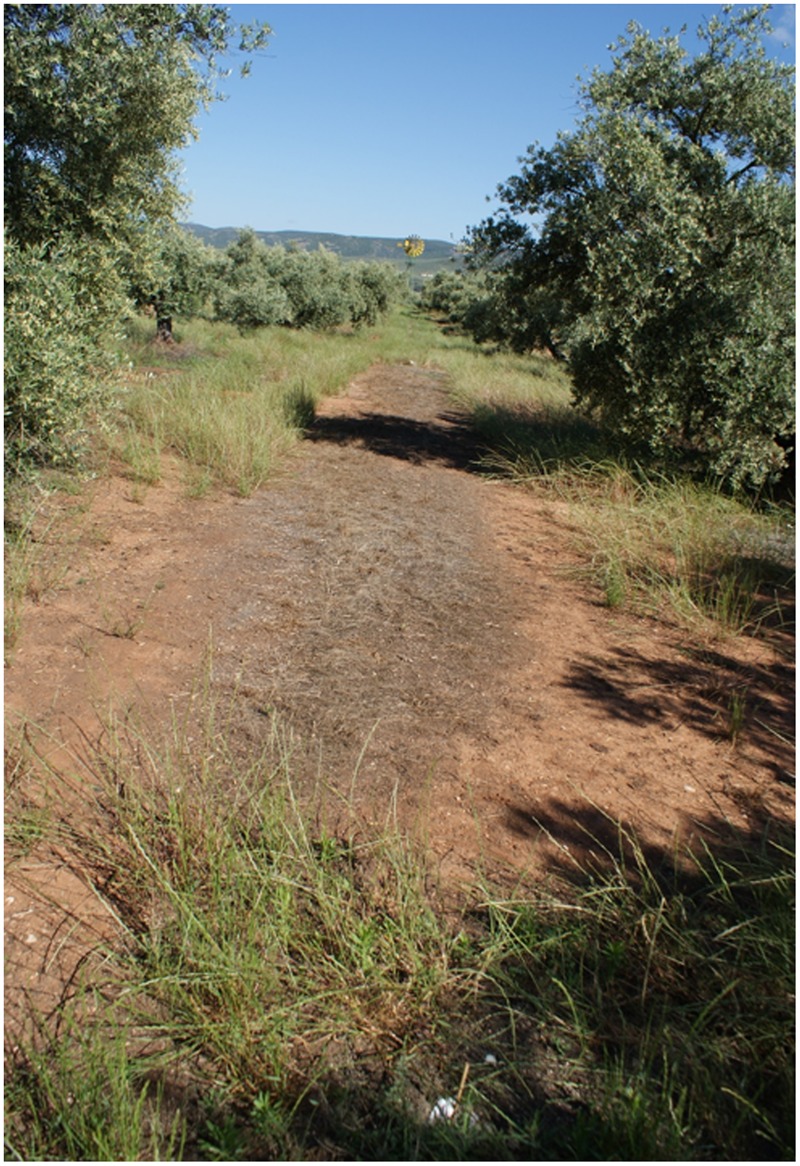
**Rigid ryegrass treated with 50 g ai ha^-1^ (grams of active ingredient per hectare) + 1800 g ae ha^-1^ (grams of acid equivalent per hectare) of flazasulfuron and glyphosate at early post-emergence and measured 60 days after treatment**.

The abundance of the RG biotype of rigid ryegrass in the original olive grove was potentially large, as denoted by the high soil cover measured in untreated plots, with mean values of 80 and 85% in years 1 and 2, respectively (**Figure 6**). *In situ* responses of the RG biotype to the different chemical treatments tested indicated that herbicide applications at the later growth stage tended to be less effective in terms of immediate effects on population size than earlier applications, with the exception of glufosinate (**Table 4**). Glyphosate-only applications at both early post-emergence and full heading led to a significant lower control effectiveness than alternative treatments within each growth stage (**Figure 7**), with the exception of full heading during the first study year, in which glyphosate effects did not differ from those of clethodim, quizalofop, and diquat (**Table 4**). These results confirm farmer’s claims regarding their inability to adequately control the local biotype of rigid ryegrass using glyphosate-only treatments. Removal of at least 85% of the RG biotype, a minimum threshold for satisfactory control, resulted from three treatments at early post-emergence, cycloxydim, flazasulfuron and flazasulfuron + glyphosate (**Figure 8**), and from the three treatments tested at tillering. At full heading, however, only glufosinate showed this effectiveness level in the second study year (**Table 4**).

**Table 4:** Effectiveness in removing the standing population and in reducing the soil seed bank of the different herbicide treatments that were tested during 2 years for *in situ* control of the R biotype of rigid ryegrass.

Treatment^a^	Effectiveness in removing the standing population (Soil cover reduction relative to untreated plots, %)^b^		Effectiveness in reducing the soil seed bank (mean seed bank reduction relative to untreated plots, %)^c^
	2013–2014	2014–2015	
**Early post-emergence**			
Glyphosate	33.7 E	31.7 D	60.5
Clethodim	65.3 D	75.0 C	91.4
Cycloxydim	88.7 B	90.0 B	100
Flazasulfuron	87.0 B	95.0 A	100
Flazasulfuron + glyphosate	97.0 A	100.0 A	100
Quizalofop-*p*-ethyl	82.0 C	78.3 C	92.8
**Tillering**			
Clethodim + glyphosate	87.0 B	88.7 B	97.1
Cycloxydim + glyphosate	92.0 A	91.7 A	100
Quizalofop-*p*-ethyl + glyphosate	87.0 B	85.0 C	95.6
**Full heading**			
Glyphosate	17.0 C	16.7 E	61.5
Glufosinate	83.7 A	88.3 A	99.5
Clethodim	23.7 BC	41.7 C	100
Cycloxydim	37.0 B	46.7 B	100
Quizalofop-*p*-ethyl	25.3 BC	31.7 D	100
Diquat	29.7 BC	35.0 D	50.9

*^a^Different herbicides were applied at three different growth stages. ^b^Effectiveness at the standing population level was measured 60 days after treatment as soil cover of rigid ryegrass relative to untreated plots. ^c^Effectiveness at the soil seed bank level was calculated as the product of mean cover reduction by mean seed set of plants surviving treatments. For each column, mean percentages of cover reduction followed by the same letter are not statistically different according to the Tukey HSD test following ANOVA.*

As expected, high mean germination percentages, 78–83%, were recorded from floret units of mature inflorescences of untreated plants of the RG biotype of rigid ryegrass (**Table 5**), this supporting germination percentage of floret units from mature inflorescences, following after-ripening, as being an adequate proxy for the seed set. A significant interaction (*p* < 0.01) between treatment and year was found for each growth stage, except for tiller stage, so one-way ANOVAs were conducted for each study year to test for treatment effects. Compared to untreated plants, a significantly lower mean seed set was found in RG plants surviving *in situ* the different herbicide treatments, including glyphosate-only applications, although the seed set was clearly dependent on herbicide identity (**Table 5**). The herbicides cycloxydim, which, however, is currently not authorized for use in olive groves, and flazasulfuron, either alone or in a mixture with glyphosate, prevented development of mature seeds in surviving treated plants irrespective of growth stage at treatment. Interestingly, clethodim and quizalofop-*p*-ethyl only prevented seed production in treatments at full heading, whereas at earlier growth stages, these herbicides, or their mixtures with glyphosate, did not fully prevent seed maturation (**Table 5**). Glyphosate-only applications tended to be among the treatments penalizing fewer seed sets of surviving plants, although seed set effects of quizalofop at early post-emergence, and diquat at full heading, did not differ significantly from glyphosate effects at the respective growth stages (**Table 5**). Evaluation of treatment effectiveness in terms of ability to reduce the soil seed bank, rather than on the basis of immediate effects on current population size, led to some contrasting conclusions (**Table 4**). With the exception of glyphosate-only treatments, and diquat at full heading, the different treatments tested appeared to be highly effective in reducing the soil seed bank (>90%). In addition, the results suggest that, with the referred exceptions of glyphosate and diquat, high effectiveness in seed bank reduction was generally achieved also at the most advanced growth stage (**Table 4**).

**Table 5:** Mean percentage seed set of rigid ryegrass plants of the R biotype surviving different herbicide treatments applied at three growth stages in field assays during two study years.

Treatment^a^	Mean seed set (%)^b^	
	2013–2014	2014–2015
**Early post-emergence**		
Untreated	82.0 A	78.0 A
Glyphosate	38.0 B	56.0 B
Clethodim	28.0 B	18.0 C
Quizalofop-*p*-ethyl	32.0 B	26.0 C
**Tillering**		
Untreated	82.0 A	78.0 A
Clethodim + glyphosate	26.0 B	12.0 B
Quizalofop-*p*-ethyl + glyphosate	28.0 B	22.0 B
**Full heading**		
Untreated	78.0 A	83.3 A
Glyphosate	26.0 C	48.0 B
Glufosinate	6.0 D	<0.1 C
Diquat	52.0 B	64.0 AB

*^a^Different herbicides were applied at three different growth stages in field assays.*

*^b^Mean seed set was measured by percentage germination recorded in random samples of floret units from mature inflorescences of rigid ryegrass plants of the R biotype surviving the herbicide treatments. For each year, a random sample of 50 floret units was tested per experimental unit. For treatments showing non-zero response within each growth stage ANOVA was carried out accounting for the experimental design of field assays to test for treatment and year effects. The interaction was significant except for tillering and thus one-way ANOVAs for each year were carried out and *post hoc* Tukey tests were used to separate mean percentages. For each column and within each growth stage, treatments with the same letter did not lead to significant differences.*

**FIGURE 9:**
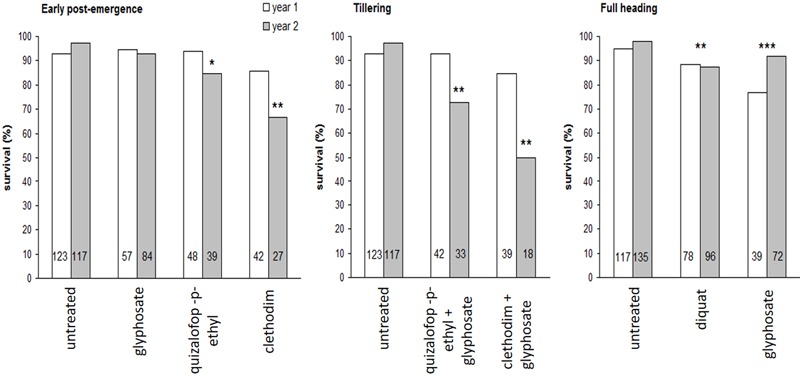
**Frequency of the R phenotype in the progeny of rigid ryegrass plants of the R biotype surviving different herbicide treatments applied at three growth stages in field assays, during the two study years.** Mean values are given. Linear mixed models with binomial error distribution and logit link function were defined taking into account the experimental design to test for treatment and year effects. Asterisks indicate a significantly lower survival rate compared to untreated plants (the reference class), i.e., the corresponding parameter associated to a given treatment in the linear component of the model significantly different from 0. For early post-emergence and tillering stages a significant treatment by year interaction was found whereas for full heading only treatment effects were significant and thus they are indicated irrespective of years. For each case, the total number of plants exposed to the glyphosate treatment is indicated within bars. ^∗^0.5 > *p* > 0.01; ^∗∗^0.01 > *p* > 0.001; ^∗∗∗^*p* < 0.001.

Treatment of progeny seedlings with glyphosate at the labeled field rate (720 g ae ha^-1^) using the laboratory spray chamber was clearly useful to separate glyphosate-resistant and susceptible phenotypes (**Figure 1**). The herbicide treatment applied “*in situ*” to the RG biotype showed a significant effect on the frequency of the resistant phenotype among the progeny of surviving plants (**Figure 9**). The frequency of the resistant phenotype within the RG biotype, as evaluated by the survival response to glyphosate of the progeny of untreated plants, was 95–96% (**Figure 9**). Clethodim (66.7%) and quizalofop-*p*-ethyl (84.6%) applied at early post-emergence in the second study year, clethodim + glyphosate (50.0%) and quizalofop-*p*-ethyl+glyphosate (72.7%) at tillering in the second study year, and diquat (mean 87.9%) and glyphosate (mean 86.3%) at full heading in both study years significantly lowered the frequency of the resistant phenotype in the progeny of surviving plants (**Figure 9**). While the fraction of glyphosate-resistant seeds produced by the plants surviving glyphosate treatment, and the untreated plants did not differ at early post-emergence, as expected, this fraction was, significantly lower for plants treated at the most advanced growth stage, as previously stated.

## Discussion

Spain is the world’s leading olive oil and table olive producer with a crop area of more than 2.5 million hectares in 2014, 62% of which is distributed in the southern region of Andalusia (Anonymous, 2014). Traditionally, weed control in olive groves had been based on deep-soil plowing, a management practice prone to causing severe soil erosion and fertility loss problems. Today, the soil management system mainly consists of cover crops that allow erosion reduction and a greater availability of moisture and nutrients to the crop plants. Currently, rigid ryegrass, either purposely sown or favoring its dominance in the weedy vegetation, is the preferred plant species to be used as a cover crop. Although the annual cycle of *L. rigidum* ends at early summer, farmers have the habit of applying selective herbicides after the first spring rains in order to stop weed growth and preventing the build-up of the soil seed bank. Since 1975, the most frequently used active substances were simazine and diuron (PSII herbicides), MCPA and fluroxypyr (auxinic inhibitor herbicides), among others. In 1990, however, glyphosate was commercialized for weed control in olive groves with immediate acceptance by farmers, making it an indispensable tool in perennial crop systems. Since the year 2000, farmers have sometimes used it at least twice within the same cultivation cycle without any additional alternative and/or IWM (Integrated Weed Management), which led to the emergence of glyphosate-resistant weed populations at the beginning of the first decade of the 21st century. In this work, for the first time, a detailed study has been carried out on the mechanisms behind glyphosate resistance in a *L. rigidum* biotype from Jaén province, Andalusia, with an emphasis on physiological, biochemical, and molecular bases, as well as on alternative chemical control options, both at the standing population and seed bank levels.

Dose-response assays demonstrated significantly higher LD_50_ and GR_50_ values for the RG biotype compared to a susceptible population (**Figure 1** and **Table 2**). These values were comparable to previous reports for other glyphosate-resistant rigid ryegrass biotypes (Adu-Yeboah et al., 2014; Fernandez et al., 2015, 2016).

In accordance with the differential behavior observed in dose-response assays, a contrasting pattern was found between RG and SG biotypes in shikimate accumulation in leaves following exposure to glyphosate. The lower shikimate accumulation observed in the RG biotype compared to the SG one (**Figure 2**) was consistent with the lower impact on the former, in terms of growth reduction and mortality, of increased rates of glyphosate. Low GR_50_ and LD_50_ values can result from an increased inhibition of EPSPS activity leading to a greater accumulation of shikimic acid (Gaines et al., 2010; Fernandez et al., 2015; Alcántara-de la Cruz et al., 2016a). However, the high levels of resistance to glyphosate and low shikimic acid accumulation in leaves exhibited by the RG biotype may also result from the addition of more than one NTSR and/or TSR mechanism, as has been shown in several grass weed species (Michitte et al., 2007; de Carvalho et al., 2012; Fernandez et al., 2015). In fact, relatively low levels of shikimate in leaves of glyphosate-resistant plants are not necessarily evidence of TSR mechanisms as this behavior has also been documented in cases of reduced foliar absorption, which leads to insufficient amounts of glyphosate in the target-site (Perez-Jones et al., 2007; Cruz-Hipolito et al., 2011; Alarcón-Reverte et al., 2015; Kleinman and Rubin, 2016).

Herbicide metabolism can be an effective NTSR mechanism (Saroha et al., 1998; Duke, 2012; Délye, 2013). However, a high proportion of glyphosate was found to remain un-metabolized in treated leaves of plants of both the RG and SG biotypes. Thus, metabolism does not appear to play any role in glyphosate resistance in the RG biotype. This result is consistent with previous studies in other glyphosate-resistant rigid ryegrass populations that did not demonstrate any contribution of metabolism to resistance (Fernandez et al., 2015). In fact, glyphosate metabolism does not seem to be a frequent resistance mechanism (Duke, 2011) and, to date, only sourgrass (de Carvalho et al., 2012), horseweed (Gonzalez-Torralva et al., 2012), and ragweed parthenium (Bracamonte et al., 2016) have been described as species able to transform glyphosate into non-toxic compounds.

Increased EPSPS enzyme activity is a plausible TSR mechanism in conferring glyphosate resistance. However, no differences were apparent between RG and SG biotypes either in EPSPS specific activity in the absence of glyphosate or in its inhibition response to glyphosate (I_50_). Thus, this relationship between EPSPS basal activity and resistance to glyphosate, which has been shown to result from EPSPS gene overexpression in some *Lolium* species (Yu et al., 2007; Dayan et al., 2012; Salas et al., 2012), is not present in the studied RG biotype. In addition, the RG biotype of rigid ryegrass did not reveal any mutation at position Pro-106 in the EPSP synthase gene, a point mutation known to endow glyphosate resistance to populations of rigid ryegrass from Australia (Wakelin and Preston, 2006) and France (Fernandez et al., 2015), and to other glyphosate-resistant weeds including the closely related species Italian ryegrass (Perez-Jones et al., 2007) and perennial ryegrass (Ghanizadeh et al., 2015), and also goosegrass (Baerson et al., 2002b). Thus, results indicate that TSR is not operating as a causal mechanism of glyphosate resistance in the studied RG biotype.

Overall, the results are in line with previous evidence of reduced uptake and translocation from other glyphosate-resistant weed populations, including rigid ryegrass (Bostamam et al., 2012; Adu-Yeboah et al., 2014; Fernandez et al., 2015), and Italian ryegrass (Michitte et al., 2007). Although impaired translocation does not occur in all glyphosate-resistant weeds, it has been considered as being the most common glyphosate resistance mechanism (Nguyen et al., 2015). Thus, it is plausible to assume that reduced uptake and translocation are the primary causal mechanisms of glyphosate resistance in the studied RG biotype of rigid ryegrass. Lower glyphosate uptake in the RG compared to SG biotype could be explained by structural differences in outer leaf surfaces (Shepherd and Griffiths, 2006; Rojano-Delgado et al., 2012; Alcántara-de la Cruz et al., 2016a), while reduced translocation could result from increased retention of glyphosate in the tips of treated leaves (de Carvalho et al., 2012; Gonzalez-Torralva et al., 2012, 2014; Adu-Yeboah et al., 2014; Bracamonte et al., 2016).

Chemical alternatives tested for *in situ* control of the RG biotype showed contrasting effects on the standing population and on the soil seed bank. Overall, treatment effectiveness was higher in terms of reduction in the contribution to the seed bank than in terms of removing the standing population (**Table 4**). This was a consequence of the markedly detrimental effect of most treatments on the seed set of surviving plants (**Table 5**). In particular, cycloxydim, flazasulfuron, and quizalofop-*p*-ethyl fully prevented seed maturation. The effect of growth stage on the ability of treatments to remove the standing population was clearly evident, with lower effectiveness at more advanced stages, a well-known, general response in chemical weed control. However, although our experimental setup did not allow for directly testing growth stage effects, treatment effects on the mean seed set of surviving plants were apparently unrelated to growth stage (**Table 5**). It should be noted that the realized contribution to the soil seed bank of plants surviving the treatments is not only determined by seed set but also by the number of florets they produced. As long as we did not measure the latter, our results represent upper estimates of the potential contribution to the soil seed bank of surviving plants, i.e., they assume that floret production was not lowered by the herbicidal treatments compared to untreated plants. Any detrimental effect of treatments on floret production would thus reinforce our findings of higher treatment effectiveness in reducing the soil seed bank than in removing the standing population.

Results of this study, focusing on the *in situ* long-term fate of the rigid ryegrass RG biotype, rather than only on immediate removal of the standing population, on which doses recommended in the product labels are based, suggest that there is a potential for implementing reduced doses of the most effective herbicide treatments. Reduced doses have been, however, associated with the rapid evolution of NTSR in outcrossing weed species like rigid ryegrass (Neve and Powles, 2005). In these cases, low detrimental doses can select for different traits conferring individually low levels of resistance but leading to increasingly resistant phenotypes through their progressive recombination allowed by outcrossing. Nevertheless, doses below labeled rates can still be recommended if target species maintain a high susceptibility (Kudsk, 2014). Reducing doses while keeping effectiveness high and alternating herbicides with contrasting modes of action can thus be a sustainable practice for management of resistance-prone rigid ryegrass populations in olive orchards in the study region.

Evaluation of the effects of “*in situ*” control treatments on the frequency of the resistant phenotype in the progeny of surviving plants indicated that most treatments significantly lowered this frequency compared to the original resistant population (i.e., a mean value of 95.5% in the progeny of untreated maternal plants), including, somewhat surprisingly, glyphosate applied at full heading. These results suggest a greater detrimental effect of these alternative herbicides, and of glyphosate applied at later growth stages, on plants of the glyphosate-resistant phenotype compared to susceptible plants within the RG population. Expression of resistance early in the plant life cycle could be at the expense of plant fitness under stressful conditions experienced at more advanced stages. Trade-offs between resistance and other functional traits have been recognized in herbicide-resistant weeds (Vila-Aiub et al., 2009), and differences in plant architecture, flowering phenology, seed dormancy depth or germination requirements, competitive ability, or resistance to diseases or pests, could explain the apparent fitness costs incurred by plants of the resistant phenotype under late stage glyphosate applications. Further research is needed to establish the existence of any such trade-offs within the RG biotype. Trade-offs can be exploited for implementing fitness-reducing management options targeting the resistant phenotype in the studied rigid ryegrass population (Vila-Aiub et al., 2005; Pedersen et al., 2007).

## Author Contributions

PF-M, and RDP performed the plant dose-response assays and the shikimic acid accumulation study; PF-M, and RDP carried out the EPSPS activity assays; PF-M and RDP did the ^14^C-glyphosate absorption/translocation and metabolism study; PF-M, and RDP performed the EPSP synthase gene sequencing; FB and PF-M carried out field experiments and FB performed germination assays and data analyses. PF-M, FB, and RDP equally contributed to writing the paper.

## Conflict of Interest Statement

The authors declare that the research was conducted in the absence of any commercial or financial relationships that could be construed as a potential conflict of interest.
